# Correlates of diabetic polyneuropathy of the elderly in Sub-Saharan Africa

**DOI:** 10.1371/journal.pone.0240602

**Published:** 2020-10-29

**Authors:** Nadine Simo, Callixte Kuate-Tegueu, Steve Ngankou-Tchankeu, Jacques Doumbe, Youssoufa Maiga, Matteo Cesari, Jean-François Dartigues, Andre-Pascal Kengne, Maturin Tabue-Teguo

**Affiliations:** 1 CHU de Guadeloupe, Equipe LAMIA, Université des Antilles, Fouillole, Guadeloupe; 2 Faculty of Medicine and Biomedical Sciences, The University of Yaoundé 1, Yaounde, Cameroon; 3 Institut Supérieur des Sciences de la Santé, Université des Montagnes, Bangangté, Cameroun; 4 Faculty of Medicine and Pharmaceutical Sciences, University of Douala, Douala, Cameroon; 5 Service de Neurologie, Hôpital Gabriel Toure (CHU), Bamako, Mali; 6 Geriatric Unit, Fondazione IRCCS Ca’ Granda Ospedale Maggiore Policlinico, Milano, Italy; 7 INSERM U 1219, Université de Bordeaux, Bordeaux, France; 8 South African Medical Research Council of South Africa, Cape Town, South Africa; Providence Care Hospital, CANADA

## Abstract

**Background:**

Diabetic polyneuropathy is associated with significant physical disability among older adults. However, their frequency and correlates are not well known in the older adults in Sub-Saharan-Africa. The objectives were to evaluate the hospital-based prevalence of diabetic polyneuropathy and identify its correlates in older adults.

**Methods:**

Over a period of 5 months, a cross-sectional survey was carried out at Douala Laquintinie Hospital (DLH), a main reference hospital in Douala, the economic capital of Cameroon. Participants in our study group comprised all patients with type 2 diabetes, whatever the reason for their reporting to the hospital. Diabetic Polyneuropathy was defined according to a Diabetic Neuropathy Examination score > 3/16.

**Results:**

A total of 159 older adults with diabetes were examined during this recruitment period, among whom 106 (66.7%) were women. The mean age was 68.3 ± 6.5 years. Diabetes median duration was 108 months. For all patients assessed using the Diabetic Neuropathy Examination score, polyneuropathy was reported in 31.4%; among them, polyneuropathy proved symptomatic in 78% of them. Correlates of polyneuropathy were glycated hemoglobin (p = 0.049), HIV infection (p = 0.031) and albuminuria (p< 0.001), even after adjustment for age, gender and duration of diabetes.

**Conclusion:**

A third of older adults with diabetes who visited our hospital were diagnosed with prevalent diabetes-related polyneuropathy. It shows that early detection is required through routine screening and regular follow-up examinations in order to reduce the risk of disability and improve the quality of life in elderly diabetics.

## Background

As a worldwide disease, diabetes is developing everywhere with no discrimination in an ever-increasing number of patients. Older adults among them are part of our study. The International Diabetes Federation (IDF) mentions the fact that the number of diabetic people over the world will rise from 415 million in 2015 to 642 million in 2040 [1–3]. The burden of diabetes in the older population is a crucial health issue for African countries [4–6]. It is estimated that 9 million Africans aged 55 years and older suffered from diabetes in 2015 [7]. Polyneuropathy is an important factor of disability in the elderly [8]. In developed countries, the most common cause of polyneuropathy is diabetes mellitus [9]. Focal or diffuse Diabetic Neuropathy (DN) is identified when patients with diabetes show pain and peripheral nerve dysfunction. The diagnosis implies dismissing other potential etiologies [2, 10]. The most classic form of DN [11, 12] is chronic sensorimotor diabetic polyneuropathy (DPN). In the larger group of sensorimotor DPN, distal symmetrical polyneuropathy (DSP) is the most common type of diabetic neuropathy [13]. Patients suffer pain, sensory disturbance, motor dysfunction, ulcers and gangrene. Polyneuropathy is a disabling disease which has a negative impact on a person’s quality of life [14]. It can provoke ulcer lesions to the feet, sometimes requiring lower limb amputations at the worst [15, 16]. Diabetic polyneuropathy is associated with significant physical disability within the elderly population [17, 18]. Among individuals with diabetes aged 60 years and more, 32% of women and 15% of men reported inability to walk one-fourth of a mile, climb stairs or perform housework; this, in comparison with 14% of women and 8% of men without diabetes. Among women, in particular, diabetes was associated with slower walking speed [19], inferior lower-extremity function, decreased balance and higher risk of falling [8, 20].

Despite the fact that diabetes and diabetic neuropathy are major and continually increasing in Africa, no data are available from this region of the world. Data from other countries and regions are not applicable to Cameroon and Sub Saharan Africa. Yet, ad hoc data are required given 1) the sociocultural and clinical differences, and 2) the emergency we are facing and the consequences due to these conditions. The elderly in Africa are undeniably at higher risk of developing polyneuropathy [9]. They are therefore at higher risk of subsequent falls and injuries. Because an increasing part of the population is over 50 years of age, especially in developing countries like Cameroon, it is important to identify the disease in this setting and then screen for correlated risk factors. This cross-sectional study was thus designed to investigate peripheral neuropathy in Cameroon’s population of older adults with diabetes. The objectives were to evaluate the hospital-based prevalence of diabetic polyneuropathy and identify its correlates in elderly patients.

## Method

### Study population

The cross-sectional study was conducted in the endocrinology, diabetes and obesity unit of DLH. The study was approved by the Institutional Ethic committee of “Université des Montagnes” Number 2013/003/CIE-UdM/Pr. A written and informed consent was obtained from all participants prior to their inclusion. A detailed description of the methodology used for the study is available elsewhere [21]. To proceed, we included all elderly diabetic patients (age ≥ 60 years) who consented to participate in the study over a 5-month period, from February to June 2013. We excluded patients with severe hearing and/or speech deficiency. Before our test, we designed a questionnaire for our compliant patients in order to collect preselected data, such as socio-demographic details, personal history, past medical information and drug taking. DPN symptoms were then examined under two authenticated criteria: Diabetic Neuropathy Symptom (DNS) score [22] and DN4 score [23]. The DNS score comprised four questions about some indications such as unsteadiness in walking, pain, paresthesia and numbness. The scale ran from 0 for absence of symptom, to maximum 4 for presence of DPN symptoms. The DN4 score included 4 questions on a scale of 10 at most. Neuropathic pain was assessed for a DN4 score ≥ 4/10. Each participant was thoroughly examined. A trained final year medical student was in charge of the examination. The specific criteria and grading involved in the Diabetic Neuropathy Examination (DNE) score [22] were applied, in particular two items relating to muscle strength, five items on reflexes and one on responsiveness. Out of a total of 8 items—each graded from 0 to 2—for a total DNE score of 16, a DNE score ≥ 3 was considered positive for DPN. All patients were also assessed under the Semmes-Weinstein monofilament test. The examination results were supervised by a consultant neurologist in a randomly selected sub-sample of participants.

### Other variables

Recently, other investigations relating to Diabetic Neuropathy Symptoms were performed. Specifically, there were tests on glycated hemoglobin, fasting blood glucose, albuminuria, serology for viral Hepatitis B and C, HIV, serum creatinine level and creatinine clearance according to the simplified Modification of Diet in Renal Disease (MDRD) equation [24].

### Applied definitions [21]

- **Polyneuropathy:** the case of an older adults suffering from diabetes, tested through DNE (Diabetic Neuropathy Examination) with a score > 3/16.
- **Symptomatic polyneuropathy:** the case of an older adults with polyneuropathy and a DNS (Diabetic Neuropathy Symptom) score ≥ 1/4.
- **Painful polyneuropathy:** the case of an older adults with polyneuropathy symptoms and consequent pain with a DN4 score > 4/10.
- **Asymptomatic polyneuropathy:** the case of an older adults with polyneuropathy and a DNS (Diabetic Neuropathy Symptom) score of 0/4.

#### Statistical analysis

Chi-squared tests and t tests were used to describe the categorical and continuous characteristics of the study sample respectively. We estimated the prevalence of diabetic polyneuropathy. Logistic regression models were performed to categorize independent polyneuropathy correlates. We developed models to account for the results about diabetic polyneuropathy in the entire elderly population of the study. *P*-values < 0.05 were considered statistically significant and 95% confidence intervals (95% CI) are provided. All analyses were performed using STATA^®^ Version 12 software (Stata Corporation, College Station, Texas).

## Results

A total of 159 older adults with diabetes were examined during the recruitment period, of whom 106 (66.7%) were women (S1 Annexe). The main features characterizing the elderly participants in our study are shown in Table 1. The mean age was 68.3 ± 6.5 years. Among the patients, 58.5% were less than 70 years old. There was no statistical difference between older and less old patients. The median duration of diagnosed diabetes was 108 months (25^th^-75^th^ percentiles: 42–180).

**Table 1:** Baseline characteristics of elderly diabetic patients (age ≥ 60 years) screened for polyneuropathy (n = 159).

Characteristics	Total number of patients (n = 159)	Polyneuropathy (n = 50)
Mean age ± SD, years	68.35 ± 6.5	68.76 ± 6.4
Females, n (%)	106 (66.6)	35 (70)
Living in Urban area, n (%)	153 (96.2)	45 (91.8)
Housewives	122 (40)	24 (48)
Traders	61 (20)	10 (20)
Retired	43 (14)	1 (2)
Civil servants	28 (9)	2 (4)
Farmers	25 (8)	6 (12)
Type 2 Diabetes, n (%)	159 (100)	50 (100)
Oral anti diabetics	125 (78.6)	40 (80)
Insulin	19 (11.9)	7 (14)
Diet only	10 (6.3)	3 (6)
Not on any treatment	5 (3.1)	0
High Blood Pressure,	93 (58.5)	33 (66)
Alcohol consumption	13 (8.2)	5 (10)
HIV infection	10 (6.3)	8 (16)
Positive for anti-HCV antibodies	12 (7.5)	7 (14)
Positive for HBV surface antigens	10 (6.3)	4 (8)
History of anti-tuberculosis treatment	6 (3.8)	3 (6)
Tobacco consumption	5 (3.1)	1 (2)
Fasting blood glucose (mean ± SD) (g/l)	1.98 ± 0.99	2.34 ± 1.07
Glycosylated hemoglobin (%)	9.07 ± 1.12	9.63 ± 1.16
Height > 1.7meters (N; %)	71 (44.6)	26 (52)
BMI > = 25 kg/m2	91 (57.2)	24 (48)
HbA1C > 7.5%	148 (93)	50 (100)
Diabetes duration > 10 years	79 (49.7)	29 (58)
Albuminuria	24 (15.1)	20 (40)

HIV: Human Immune deficiency virus, HCV: Hepatitis C virus, HBV: Hepatitis B virus, SD: standard deviation

Polyneuropathy symptoms were found in 101 (63.5%) patients. The most frequent signs were: walking instability (52.2%), burning sensation in the feet (49%), prickling (43.4%) and numbness in the lower limbs (43.4%). We found 58 (73.4%) patients with neuropathic pain and 21 (26.6%) of them showing nociceptive pain, this on a total of 79 patients submitted to the DN4 scale.

Normal muscle capacity in femoris quadriceps in 64.8% of the patients had decreased in 35.2% of them. Normal anterior tibialis in 65.4% of the patients had decreased in 34.6% of them. Triceps surae reflex which was normal in 69.8% of the patients had decreased in 30.2% of them. Sensitivity to touch at the index had decreased in 13.8% of the patients and disappeared in 1.2% of them. Sensitivity to pinpricks had decreased in 20.7% of the patients and disappeared in 3.1% of them. Sensitivity to touch at the toe had decreased in 22% of the patients and disappeared in 1.2% of them. Vibration perception at the great toe had decreased in 25.4% of the patients and disappeared in 14.5% of them. Sensitivity to joint position at the big toe had decreased in 7.5% of the patients and disappeared in 2% of them.

Of all patients who were controlled through Diabetic Neuropathy Examination (DNE) score, 50 (31.4%) showed polyneuropathy clinical signs with symptomatic polyneuropathy in 39 (78%) patients. Within the DN4 scale score, painful polyneuropathy was detected in 32 out of 50 (64%) patients. For all patients tested with the monofilament test, polyneuropathy was present in 47 (29.5%) of them. According to regression models analyses, which involved a single variable and covered the overall study population, the signs mentioned hereafter were associated with polyneuropathy: urban residence (p = 0.03), higher fasting blood glucose at inclusion (p = 0.002), glycated hemoglobin level above 7.5% (p < 0.0001), positive HIV serology (p = 0.004), presence of albuminuria (p = 0.0001), known chronic kidney disease (p = 0 003) and hepatitis C virus infection (p = 0.047) (Table 2). Patients who had had diabetes for more than 10 years, were more predisposed to having polyneuropathy in comparison to those with diabetes for less than 10 years; but the difference was not substantial (p = 0.051). In multivariate logistic regression models, significant polyneuropathy correlates were glycated hemoglobin level above 7.5% (OR = 2.2 [1.0–5.1], p = 0.049), HIV infection (OR = 7.1 [1.2–43.0], p = 0.031) and presence of albuminuria (OR = 12.7 [3.8–42.0], p = 0.0001) (see Table 2).

**Table 2:** Univariable and multivariable analysis of factors associated with diabetic polyneuropathy in elderly patients in Douala-Cameroon (n = 159).

	Univariate analysis		Multivariate analysis (Adjusted for sex, age and duration of diabetes)	
Factor	OR (95% CI)	P-value	OR (95% CI)	P-value
Urban residence	5.3 (1.0–28)	0.050	-	NA
Duration of diabetes >15 years	2.0 (1.0–4.5)	0.051	-	NA
Fasting blood glucose at inclusion	1.7 (1.2–2.4)	0.042	-	NA
Glycated hemoglobin	1.9 (1.4–2.7)	0.0001	2.2 (1.0–5.1)	**0.049**
Height >1.7m	1.8 (0.8–3.8)	0.136	-	-
Body mass index >25kg/m^2^	0.5 (0.3–1.1)	0.113	-	-
Presence of anti-HCV antibodies	3.3 (1.0–11.2)	0.047	1.6 (0.3–7.1)	0.526
Presence of anti-HIV antibodies	10.2 (2.0–49.9)	0.004	7.1 (1.2–43.0)	**0.031**
*Known Chronic renal disease	2.9 (1.4–6.0)	0.033	-	-
Chronic alcoholism	1.4 (0.4–4.5)	0.571	-	NA
Albuminuria	17.5 (5.5–55.1)	0.0001	12.7 (3.8–42.0)	**0.0001**

*Not included in multivariate analysis because of too few values;

HCV: Hepatitis C virus; HIV: Human immune deficiency virus; OR: Odds ratio; 95%CI: 95% confidence interval

## Discussion

This study involved older adults with diabetes who received treatment in a tertiary care hospital in Cameroon. Two in three patients who participated in the study showed polyneuropathy symptoms. Under DNE score, diabetic neuropathy was diagnosed in a third of the participants, proving a huge number. The key features of polyneuropathy in the elderly were: microvascular problems related to diabetes, viral infections and glycaemia abnormal values. The risk of viral infections spread to our setting could be another potential cause for the extension and acceleration of neuropathy incidence in the elderly people with diabetes.

### Polyneuropathy prevalence

Our results verified a general prevalence of diabetic polyneuropathy: 31.4% based on the DNE score and 29.5% based on the monofilament test. Whether based on the monofilament test or the DNE score, both results on diabetic polyneuropathy prevalence were mostly the same. Dismissing other possible causes, such as anti-tuberculosis treatment, HIV infection, chronic renal disease, the prevalence of diabetic polyneuropathy in our elderly patients was 31.4%. We did not find a similar hospital-based study in literature about polyneuropathy in the elderly. However, our results could compare with those from a number of reports on general adult populations with diabetes [25, 26]. Hanewinckel et al. [27] in a review on epidemiology and risk factors of chronic polyneuropathy identified 29 population-based studies. There was a large variation in reported prevalence rates across these studies (1.9 to 30.9% in the elderly), which was probably due to the diversity in assessment protocols, the definition given to polyneuropathy, the targeted populations and the study parameters. As a whole, prevalence of polyneuropathy in door-to-door survey studies from developed countries seemed higher than in studies performed in developing countries. This could be partly explained by the fact that a larger proportion of elderly people were included in studies performed in developed countries.

The prevalence of painful diabetic polyneuropathy was 20.1% in our study. No similar result in elderly patients was found in the literature. In the population at large, Vinik et al. [28, 29] reported respectively 16% and 26% for the prevalence of painful diabetic neuropathy in 1992 and 2004 studies conducted in the United Kingdom. In a community-based diabetic population in the UK, Abbot et al. used a neuropathy symptom score [30] and reported a 60% prevalence of painful diabetic neuropathy. However, there was a large disparity in the study between signs and symptoms: approximately one-quarter of the patients with no clinical neuropathy on examination presented significant painful neuropathy symptoms.

### Risk factors in polyneuropathy

In our study, we showed noticeable links between diabetic polyneuropathy and its causes such as high levels of glycated hemoglobin, HIV infection and albuminuria. The current results confirmed what had already been stated through test scores about the connection between polyneuropathy, HIV infection [31], albuminuria [32] and glycaemia [33]. Early glucose control in patients with diabetes proved fundamental to manage polyneuropathy, prevent it or reduce its effects. Our conclusions approximated those of Kong et al. [32] in associating increase in albuminuria with polyneuropathy, involving kidney pathophysiology, diabetes and micro-vascularity. It had already been stated that polyneuropathy was also related to specific diabetes micro-vascular problems like retinopathy [34]. Yet, we did not find a substantial connection between diabetic polyneuropathy and the following items: duration of diabetes, high blood pressure, hypercholesterolemia, smoking habits and body height or body mass index. This was in contrast with the findings [35] in Bangladesh, in China [33, 36] and some other reports [37–39]. The reason could have been the limited size of the sample. However, we discovered a doubled increase in risk relating to diabetes duration (OR = 2.0; 95% CI (1.0–4.5). The participants in our study being patients with type 2 diabetes, the result was not wholly representative as in the case of a complete duration of the disease. For a majority of our patients, diabetes was diagnosed at a stage of complications. Due to financial restraints and lack of social security, elderly patients did not control their blood glucose regularly. Most patients exceeded normal glucose level range. Glycemic control indicators were necessary to differentiate polyneuropathy types among our diabetic participants. Alcohol-related peripheral neuropathy is a potentially debilitating complication linked to alcoholism that results in sensory, motor and autonomic dysfunction [40]. Alcohol-related polyneuropathy is known to be of toxic origin [41–43] compared with nutritional deficiency neuropathy [40]. In our study, we did not find a meaningful statistical association between chronic alcohol consumption and polyneuropathy. This may partly be explained by the fact that diabetic patients in our settings consumed less alcohol as recommended in their treatment protocol. We may have underestimated the chronic alcoholism prevalence, due to the fact that the diagnosis criteria involved estimate rather than assessment in this particular case. The link between chronic alcoholism and polyneuropathy has still to be proven with more certainty. Along our study, we made use of DNS score, DNE score, DN4 score and monofilament test score. Our methodology was as accurate as possible in assessing polyneuropathy symptoms. We tried to be clear in our concepts and their definitions, in the ratings conducted and the results obtained. The main difficulties came from a rather limited sample. Some examples of fiber lesions, which show nerve deficit at an early stage and help determine asymptomatic PN, were few because people came late for a clinical assessment. The risk factors for diabetes polyneuropathy clearly need to be studied. The challenge is to establish the causes of diseases in the elderly population in Sub-Saharan Africa by performing a prospective follow-up study. While prevalence data are particularly relevant for health service planning, incidence data provide the basis for furthering our understanding of these diseases for implementing a real prevention of age-related conditions.

## Conclusion

The problem of diabetic polyneuropathy is major among the elderly patients with diabetes. The patients who participated in our study were assessed in the urban specialized hospital in Douala, Cameroon. We found higher polyneuropathy prevalence among the patients with albuminuria, infection with HIV and high glycosylated hemoglobin level. Previous studies have showed the damaging impact of further disablement linked to polyneuropathy among diabetic people in Cameroon. On their admission to hospital, diabetic patients presented health complications from foot ulcer and viral infections to chronic pathologies. Interventions in the form of early detection, through routine screening and regular follow-up examinations, would go a long way in reducing the burden of disability among elderly diabetics and would improve their quality of life significantly.

## Supporting information

S1 Annexe (DOCX)

S2 Annexe (DOCX)

## Abbreviations

DLH: Douala Laquintinie Hospital
DN4: Neuropathic pain diagnostic questionnaire
DNE: Diabetic Neuropathy Examination
DNS score: Diabetic Neuropathy Symptom score
DPN: Diabetic polyneuropathy
DSP: Distal symmetrical polyneuropathy
HIV: Human Immuno deficiency virus
IDF: International Diabetes Federation
MDRD: simplified Modification of Diet in Renal Disease
OR: Odds ratio
PN: Polyneuropathy
