# Supplementary material for: Correlates of diabetic polyneuropathy of the elderly in Sub-Saharan Africa
Source: PLoS One. 2020 Oct 29;15(10):e0240602. doi: 10.1371/journal.pone.0240602 (PMC7595408; doi:10.1371/journal.pone.0240602)
Supplement: S1 Annexe — (DOCX) [file pone.0240602.s001.docx]

S1 Annexe

306 participants with diabetic polyneuropathy during the study period

159 participants

Under 60 years: 147

**Flow chart**
