# Supplementary material for: Correlates of diabetic polyneuropathy of the elderly in Sub-Saharan Africa
Source: PLoS One. 2020 Oct 29;15(10):e0240602. doi: 10.1371/journal.pone.0240602 (PMC7595408; doi:10.1371/journal.pone.0240602)
Supplement: S2 Annexe — (DOCX) [file pone.0240602.s002.docx]

**S2 Annexe : Data analyzed**

-> tabulation of age

Age du |

patient | Freq. Percent Cum.

------------+-----------------------------------

60 | 19 11.95 11.95

61 | 9 5.66 17.61

62 | 7 4.40 22.01

63 | 14 8.81 30.82

64 | 9 5.66 36.48

65 | 8 5.03 41.51

66 | 7 4.40 45.91

67 | 4 2.52 48.43

68 | 9 5.66 54.09

69 | 7 4.40 58.49

70 | 11 6.92 65.41

71 | 6 3.77 69.18

72 | 5 3.14 72.33

73 | 5 3.14 75.47

74 | 9 5.66 81.13

75 | 4 2.52 83.65

76 | 5 3.14 86.79

77 | 4 2.52 89.31

78 | 3 1.89 91.19

79 | 6 3.77 94.97

80 | 3 1.89 96.86

81 | 1 0.63 97.48

83 | 1 0.63 98.11

84 | 2 1.26 99.37

87 | 1 0.63 100.00

------------+-----------------------------------

Total | 159 100.00

-> tabulation of age1

Age | Freq. Percent Cum.

------------+-----------------------------------

60-64 | 58 36.48 36.48

65-69 | 35 22.01 58.49

70-74 | 36 22.64 81.13

75-79 | 22 13.84 94.97

80+ | 8 5.03 100.00

------------+-----------------------------------

Total | 159 100.00

-> tabulation of sex

Sexe du |

patient | Freq. Percent Cum.

------------+-----------------------------------

0. Feminin | 106 66.67 66.67

1. Masculin | 53 33.33 100.00

------------+-----------------------------------

Total | 159 100.00

-> tabulation of res

Residence |

en zone |

urbaine ou |

rurale | Freq. Percent Cum.

------------+-----------------------------------

0. Urbain | 153 96.23 96.23

1. Rural | 5 3.14 99.37

. | 1 0.63 100.00

------------+-----------------------------------

Total | 159 100.00

-> tabulation of type

Type de |

diabete | Freq. Percent Cum.

------------+-----------------------------------

1. Type2 | 159 100.00 100.00

------------+-----------------------------------

Total | 159 100.00

-> tabulation of dur

duree du |

diabete |

(mois) | Freq. Percent Cum.

------------+-----------------------------------

1 | 9 5.66 5.66

2 | 3 1.89 7.55

3 | 2 1.26 8.81

6 | 1 0.63 9.43

7 | 1 0.63 10.06

8 | 1 0.63 10.69

12 | 4 2.52 13.21

24 | 8 5.03 18.24

36 | 15 9.43 27.67

48 | 7 4.40 32.08

60 | 7 4.40 36.48

72 | 7 4.40 40.88

82 | 1 0.63 41.51

84 | 4 2.52 44.03

92 | 1 0.63 44.65

96 | 6 3.77 48.43

108 | 3 1.89 50.31

120 | 19 11.95 62.26

122 | 1 0.63 62.89

132 | 4 2.52 65.41

144 | 6 3.77 69.18

156 | 4 2.52 71.70

168 | 1 0.63 72.33

180 | 6 3.77 76.10

204 | 1 0.63 76.73

240 | 11 6.92 83.65

252 | 3 1.89 85.53

253 | 1 0.63 86.16

264 | 1 0.63 86.79

276 | 1 0.63 87.42

288 | 5 3.14 90.57

300 | 1 0.63 91.19

312 | 3 1.89 93.08

360 | 6 3.77 96.86

384 | 1 0.63 97.48

420 | 2 1.26 98.74

480 | 2 1.26 100.00

------------+-----------------------------------

Total | 159 100.00

-> tabulation of dur1

Duree du |

diabete | Freq. Percent Cum.

------------+-----------------------------------

<=10ans | 99 62.26 62.26

>10ans | 60 37.74 100.00

------------+-----------------------------------

Total | 159 100.00

-> tabulation of vih

Serologie |

VIH |

positive? | Freq. Percent Cum.

-------------+-----------------------------------

0. Non | 149 93.71 93.71

1. Oui | 10 6.29 100.00

-------------+-----------------------------------

Total | 159 100.00

-> tabulation of hbv

AgHBs |

positif? | Freq. Percent Cum.

-------------+-----------------------------------

0. Non | 149 93.71 93.71

1. Oui | 10 6.29 100.00

-------------+-----------------------------------

Total | 159 100.00

-> tabulation of hcv

Serologie |

HVC |

positive? | Freq. Percent Cum.

-------------+-----------------------------------

0. Non | 147 92.45 92.45

1. Oui | 12 7.55 100.00

-------------+-----------------------------------

Total | 159 100.00

-> tabulation of trai

Traitement |

antidiabetique |

actuel | Freq. Percent Cum.

---------------+-----------------------------------

0. Aucun | 5 3.14 3.14

1. ADO | 8 5.03 8.18

2. RegimAlim | 10 6.29 14.47

3. Insul+Regim | 17 10.69 25.16

4. Insuline | 2 1.26 26.42

5. ADO+Regim | 117 73.58 100.00

---------------+-----------------------------------

Total | 159 100.00

-> tabulation of irc

Patient |

connu IRC? | Freq. Percent Cum.

-------------+-----------------------------------

0. Non | 155 97.48 97.48

1. Oui | 3 1.89 99.37

2. NeSaisPas | 1 0.63 100.00

-------------+-----------------------------------

Total | 159 100.00

-> tabulation of hta

Antecedant |

d'HTA du |

patient | Freq. Percent Cum.

-------------+-----------------------------------

0. Non | 66 41.51 41.51

1. Oui | 93 58.49 100.00

-------------+-----------------------------------

Total | 159 100.00

-> tabulation of alc

Consommatio |

n d'alcool? | Freq. Percent Cum.

------------+-----------------------------------

0. Non | 146 91.82 91.82

1. Oui | 13 8.18 100.00

------------+-----------------------------------

Total | 159 100.00

-> tabulation of tab

Consommatio |

n de Tabac? | Freq. Percent Cum.

------------+-----------------------------------

0. Non | 154 96.86 96.86

1. Oui | 5 3.14 100.00

------------+-----------------------------------

Total | 159 100.00

-> tabulation of tbc

traitement |

antitubercul |

eux passe/en |

cour? | Freq. Percent Cum.

-------------+-----------------------------------

0. Non | 153 96.23 96.23

1. Oui | 6 3.77 100.00

-------------+-----------------------------------

Total | 159 100.00

-> tabulation of ins

instabilite |

a la marche | Freq. Percent Cum.

------------+-----------------------------------

0. Non | 76 47.80 47.80

1. Oui | 83 52.20 100.00

------------+-----------------------------------

Total | 159 100.00

-> tabulation of tai

Taille du |

patient | Freq. Percent Cum.

------------+-----------------------------------

1.55 | 4 2.52 2.52

1.56 | 1 0.63 3.14

1.57 | 1 0.63 3.77

1.58 | 1 0.63 4.40

1.59 | 1 0.63 5.03

1.60 | 20 12.58 17.61

1.61 | 3 1.89 19.50

1.62 | 2 1.26 20.75

1.64 | 4 2.52 23.27

1.65 | 31 19.50 42.77

1.66 | 3 1.89 44.65

1.67 | 14 8.81 53.46

1.68 | 2 1.26 54.72

1.69 | 1 0.63 55.35

1.70 | 35 22.01 77.36

1.71 | 4 2.52 79.87

1.72 | 2 1.26 81.13

1.73 | 4 2.52 83.65

1.74 | 4 2.52 86.16

1.75 | 11 6.92 93.08

1.77 | 1 0.63 93.71

1.78 | 2 1.26 94.97

1.80 | 4 2.52 97.48

1.82 | 1 0.63 98.11

1.87 | 1 0.63 98.74

1.88 | 2 1.26 100.00

------------+-----------------------------------

Total | 159 100.00

-> tabulation of imc

RECODE of |

bmi | Freq. Percent Cum.

-------------+-----------------------------------

0. <18.5 | 2 1.26 1.26

1. 18.5-24.9 | 66 41.51 42.77

2. 25-29.9 | 58 36.48 79.25

3. 30-34.9 | 25 15.72 94.97

4. >35 | 8 5.03 100.00

-------------+-----------------------------------

Total | 159 100.00

-> tabulation of imc1

Recoded IMC | Freq. Percent Cum.

------------+-----------------------------------

<25 | 68 42.77 42.77

>=25 | 91 57.23 100.00

------------+-----------------------------------

Total | 159 100.00

-> tabulation of hba1c

Hb glyquee |

des |

6derniers |

mois | Freq. Percent Cum.

------------+-----------------------------------

7.2 | 4 2.52 2.52

7.3 | 2 1.26 3.77

7.4 | 1 0.63 4.40

7.5 | 4 2.52 6.92

7.7 | 7 4.40 11.32

7.8 | 3 1.89 13.21

7.9 | 7 4.40 17.61

8.0 | 9 5.66 23.27

8.2 | 2 1.26 24.53

8.3 | 6 3.77 28.30

8.4 | 4 2.52 30.82

8.5 | 11 6.92 37.74

8.6 | 1 0.63 38.36

8.7 | 12 7.55 45.91

8.8 | 3 1.89 47.80

8.9 | 11 6.92 54.72

9.0 | 8 5.03 59.75

9.1 | 1 0.63 60.38

9.3 | 2 1.26 61.64

9.4 | 5 3.14 64.78

9.5 | 3 1.89 66.67

9.6 | 1 0.63 67.30

9.7 | 6 3.77 71.07

9.8 | 8 5.03 76.10

9.9 | 2 1.26 77.36

10.0 | 8 5.03 82.39

10.4 | 2 1.26 83.65

10.5 | 4 2.52 86.16

10.6 | 1 0.63 86.79

10.7 | 8 5.03 91.82

10.8 | 2 1.26 93.08

10.9 | 2 1.26 94.34

11.0 | 5 3.14 97.48

11.1 | 1 0.63 98.11

11.7 | 1 0.63 98.74

12.5 | 2 1.26 100.00

------------+-----------------------------------

Total | 159 100.00

-> tabulation of hba1c1

Hb glyquee |

des 6 |

derniers |

mois | Freq. Percent Cum.

------------+-----------------------------------

7.2-7.5 | 11 6.92 6.92

7.6-9 | 84 52.83 59.75

9.1-12.5 | 64 40.25 100.00

------------+-----------------------------------

Total | 159 100.00

-> tabulation of gly

Glycemie a |

jeun du |

jour | Freq. Percent Cum.

------------+-----------------------------------

0.59 | 1 0.63 0.63

0.60 | 2 1.26 1.89

0.70 | 1 0.63 2.52

0.78 | 1 0.63 3.14

0.85 | 1 0.63 3.77

0.89 | 1 0.63 4.40

0.90 | 2 1.26 5.66

0.93 | 1 0.63 6.29

0.95 | 1 0.63 6.92

0.97 | 3 1.89 8.81

0.98 | 1 0.63 9.43

0.99 | 2 1.26 10.69

1.00 | 2 1.26 11.95

1.02 | 1 0.63 12.58

1.03 | 1 0.63 13.21

1.04 | 1 0.63 13.84

1.06 | 3 1.89 15.72

1.09 | 1 0.63 16.35

1.10 | 1 0.63 16.98

1.12 | 2 1.26 18.24

1.14 | 1 0.63 18.87

1.15 | 1 0.63 19.50

1.16 | 1 0.63 20.13

1.18 | 2 1.26 21.38

1.19 | 2 1.26 22.64

1.20 | 2 1.26 23.90

1.22 | 2 1.26 25.16

1.23 | 1 0.63 25.79

1.24 | 1 0.63 26.42

1.25 | 1 0.63 27.04

1.26 | 1 0.63 27.67

1.27 | 2 1.26 28.93

1.28 | 1 0.63 29.56

1.29 | 1 0.63 30.19

1.31 | 2 1.26 31.45

1.32 | 1 0.63 32.08

1.34 | 1 0.63 32.70

1.35 | 2 1.26 33.96

1.38 | 2 1.26 35.22

1.40 | 2 1.26 36.48

1.42 | 1 0.63 37.11

1.44 | 1 0.63 37.74

1.45 | 1 0.63 38.36

1.49 | 1 0.63 38.99

1.54 | 1 0.63 39.62

1.56 | 1 0.63 40.25

1.57 | 1 0.63 40.88

1.58 | 2 1.26 42.14

1.60 | 3 1.89 44.03

1.65 | 2 1.26 45.28

1.67 | 1 0.63 45.91

1.71 | 2 1.26 47.17

1.72 | 1 0.63 47.80

1.76 | 4 2.52 50.31

1.78 | 2 1.26 51.57

1.80 | 1 0.63 52.20

1.81 | 1 0.63 52.83

1.83 | 1 0.63 53.46

1.85 | 1 0.63 54.09

1.86 | 1 0.63 54.72

1.87 | 5 3.14 57.86

1.90 | 3 1.89 59.75

1.94 | 1 0.63 60.38

1.97 | 1 0.63 61.01

2.00 | 7 4.40 65.41

2.01 | 1 0.63 66.04

2.03 | 2 1.26 67.30

2.10 | 1 0.63 67.92

2.14 | 1 0.63 68.55

2.20 | 1 0.63 69.18

2.28 | 2 1.26 70.44

2.29 | 2 1.26 71.70

2.34 | 1 0.63 72.33

2.36 | 1 0.63 72.96

2.43 | 1 0.63 73.58

2.46 | 1 0.63 74.21

2.50 | 2 1.26 75.47

2.55 | 1 0.63 76.10

2.56 | 1 0.63 76.73

2.65 | 1 0.63 77.36

2.74 | 1 0.63 77.99

2.76 | 1 0.63 78.62

2.80 | 2 1.26 79.87

2.84 | 1 0.63 80.50

2.85 | 1 0.63 81.13

2.88 | 1 0.63 81.76

2.90 | 3 1.89 83.65

2.93 | 1 0.63 84.28

2.97 | 1 0.63 84.91

3.00 | 2 1.26 86.16

3.03 | 1 0.63 86.79

3.05 | 1 0.63 87.42

3.10 | 1 0.63 88.05

3.20 | 1 0.63 88.68

3.27 | 1 0.63 89.31

3.30 | 1 0.63 89.94

3.34 | 1 0.63 90.57

3.40 | 3 1.89 92.45

3.56 | 1 0.63 93.08

3.60 | 1 0.63 93.71

3.76 | 1 0.63 94.34

3.80 | 1 0.63 94.97

3.86 | 1 0.63 95.60

4.00 | 1 0.63 96.23

4.30 | 1 0.63 96.86

4.39 | 1 0.63 97.48

4.91 | 1 0.63 98.11

5.30 | 1 0.63 98.74

5.40 | 1 0.63 99.37

5.60 | 1 0.63 100.00

------------+-----------------------------------

Total | 159 100.00

-> tabulation of creat

Creatininem |

ie des |

6derniers |

mois | Freq. Percent Cum.

------------+-----------------------------------

6.3 | 1 0.63 0.63

8.0 | 7 4.40 5.03

8.3 | 1 0.63 5.66

8.5 | 1 0.63 6.29

8.6 | 2 1.26 7.55

8.7 | 4 2.52 10.06

9.0 | 18 11.32 21.38

9.7 | 5 3.14 24.53

9.8 | 2 1.26 25.79

10.0 | 15 9.43 35.22

10.4 | 1 0.63 35.85

10.8 | 1 0.63 36.48

11.0 | 17 10.69 47.17

11.3 | 1 0.63 47.80

11.4 | 2 1.26 49.06

11.5 | 1 0.63 49.69

11.6 | 1 0.63 50.31

11.7 | 2 1.26 51.57

11.8 | 1 0.63 52.20

12.0 | 9 5.66 57.86

12.3 | 1 0.63 58.49

12.4 | 1 0.63 59.12

12.7 | 1 0.63 59.75

13.0 | 11 6.92 66.67

13.4 | 1 0.63 67.30

13.7 | 1 0.63 67.92

14.0 | 9 5.66 73.58

14.5 | 1 0.63 74.21

14.6 | 1 0.63 74.84

14.7 | 1 0.63 75.47

14.8 | 1 0.63 76.10

15.0 | 4 2.52 78.62

15.6 | 1 0.63 79.25

16.0 | 8 5.03 84.28

16.7 | 2 1.26 85.53

17.0 | 3 1.89 87.42

17.3 | 1 0.63 88.05

17.4 | 1 0.63 88.68

18.0 | 3 1.89 90.57

18.3 | 1 0.63 91.19

18.6 | 1 0.63 91.82

18.7 | 1 0.63 92.45

19.0 | 2 1.26 93.71

19.7 | 1 0.63 94.34

24.8 | 1 0.63 94.97

26.4 | 1 0.63 95.60

27.0 | 1 0.63 96.23

34.0 | 3 1.89 98.11

51.0 | 1 0.63 98.74

52.0 | 1 0.63 99.37

56.0 | 1 0.63 100.00

------------+-----------------------------------

Total | 159 100.00

-> tabulation of creat1

Creatininem |

des 6 |

derniers |

mois | Freq. Percent Cum.

------------+-----------------------------------

6.3-12.0 | 92 57.86 57.86

12.1-56.0 | 67 42.14 100.00

------------+-----------------------------------

Total | 159 100.00

-> tabulation of prot

Proteinurie |

a la BU | Freq. Percent Cum.

------------+-----------------------------------

0. Absent | 135 84.91 84.91

1. 1+ | 20 12.58 97.48

2. 2+ | 4 2.52 100.00

------------+-----------------------------------

Total | 159 100.00

-> tabulation of dn4

Score |

Douleur |

Neuropathiq |

ue en |

4questions | Freq. Percent Cum.

------------+-----------------------------------

2 | 3 1.89 1.89

3 | 2 1.26 3.14

4 | 16 10.06 13.21

5 | 27 16.98 30.19

6 | 23 14.47 44.65

7 | 7 4.40 49.06

8 | 1 0.63 49.69

. | 80 50.31 100.00

------------+-----------------------------------

Total | 159 100.00

-> tabulation of dne

Score DNE | Freq. Percent Cum.

------------+-----------------------------------

0 | 76 47.80 47.80

1 | 10 6.29 54.09

2 | 15 9.43 63.52

3 | 8 5.03 68.55

4 | 10 6.29 74.84

5 | 9 5.66 80.50

6 | 16 10.06 90.57

7 | 6 3.77 94.34

8 | 4 2.52 96.86

9 | 1 0.63 97.48

10 | 2 1.26 98.74

11 | 1 0.63 99.37

14 | 1 0.63 100.00

------------+-----------------------------------

Total | 159 100.00

-> tabulation of dns

Diabetic |

Neuropathy |

Symptom |

Score | Freq. Percent Cum.

------------+-----------------------------------

0 | 58 36.48 36.48

1 | 24 15.09 51.57

2 | 7 4.40 55.97

3 | 19 11.95 67.92

4 | 51 32.08 100.00

------------+-----------------------------------

Total | 159 100.00

. logistic dne1 age

Logistic regression Number of obs = 159

LR chi2(1) = 0.15

Prob > chi2 = 0.6946

Log likelihood = -104.24582 Pseudo R2 = 0.0007

**AGE : Non significatif**

------------------------------------------------------------------------------

dne1 | Odds Ratio Std. Err. z P>|z| [95% Conf. Interval]

-------------+----------------------------------------------------------------

age | .990038 .0252999 -0.39 0.695 .9416724 1.040888

_cons | 1.137783 1.992829 0.07 0.941 .0367433 35.23226

------------------------------------------------------------------------------

. logistic dne1 i.age1

Logistic regression Number of obs = 159

LR chi2(4) = 7.71

Prob > chi2 = 0.1028

Log likelihood = -100.46792 Pseudo R2 = 0.0370

------------------------------------------------------------------------------

dne1 | Odds Ratio Std. Err. z P>|z| [95% Conf. Interval]

-------------+----------------------------------------------------------------

age1 |

65-69 | 1.090909 .4783608 0.20 0.843 .4618904 2.576548

70-74 | .5454545 .2566423 -1.29 0.198 .2168997 1.371697

75-79 | 1.963636 .9946272 1.33 0.183 .7276253 5.299249

80+ | .2337662 .2577891 -1.32 0.188 .026922 2.029816

|

_cons | .6111111 .1653757 -1.82 0.069 .3595609 1.038647

------------------------------------------------------------------------------

. logistic dne1 i.sex

Logistic regression Number of obs = 159

LR chi2(1) = 0.67

Prob > chi2 = 0.4125

Log likelihood = -103.98708 Pseudo R2 = 0.0032

**SEXE MASCULIN : Non significatif**

------------------------------------------------------------------------------

dne1 | Odds Ratio Std. Err. z P>|z| [95% Conf. Interval]

-------------+----------------------------------------------------------------

sex |

1. Masculin | .748645 .266139 -0.81 0.415 .3729741 1.502703

_cons | .6307692 .1257983 -2.31 0.021 .4266875 .9324618

------------------------------------------------------------------------------

. logistic dne1 i.res

Logistic regression Number of obs = 158

LR chi2(1) = 4.18

Prob > chi2 = 0.0408

Log likelihood = -101.21652 Pseudo R2 = 0.0203

**RESIDENCE : Non significatif**

------------------------------------------------------------------------------

dne1 | Odds Ratio Std. Err. z P>|z| [95% Conf. Interval]

-------------+----------------------------------------------------------------

res |

1. Rural | 7.547168 8.534869 1.79 0.074 .8225938 69.24408

_cons | .53 .09005 -3.74 0.000 .3798848 .7394347

------------------------------------------------------------------------------

. logistic dne1 i.type

note: 1.type omitted because of collinearity

Logistic regression Number of obs = 159

LR chi2(0) = 0.00

Prob > chi2 = .

Log likelihood = -104.3229 Pseudo R2 = 0.0000

------------------------------------------------------------------------------

dne1 | Odds Ratio Std. Err. z P>|z| [95% Conf. Interval]

-------------+----------------------------------------------------------------

type |

1. Type2 | 1 (omitted)

_cons | .5742574 .0946086 -3.37 0.001 .4157877 .7931249

------------------------------------------------------------------------------

. logistic dne1 dur

Logistic regression Number of obs = 159

LR chi2(1) = 2.99

Prob > chi2 = 0.0836

Log likelihood = -102.82634 Pseudo R2 = 0.0143

**DUREE DU DIABETE général : non significatif**

------------------------------------------------------------------------------

dne1 | Odds Ratio Std. Err. z P>|z| [95% Conf. Interval]

-------------+----------------------------------------------------------------

dur | 1.002528 .0014686 1.72 0.085 .999654 1.005411

_cons | .4104638 .1064714 -3.43 0.001 .2468761 .6824496

------------------------------------------------------------------------------

. logistic dne1 i.dur1

Logistic regression Number of obs = 159

LR chi2(1) = 1.11

Prob > chi2 = 0.2915

Log likelihood = -103.7665 Pseudo R2 = 0.0053

**DUREE DU DIABETE > 10 ans : Non significatif**

------------------------------------------------------------------------------

dne1 | Odds Ratio Std. Err. z P>|z| [95% Conf. Interval]

-------------+----------------------------------------------------------------

dur1 |

>10ans | 1.428571 .4823962 1.06 0.291 .7370051 2.769067

_cons | .5 .1066004 -3.25 0.001 .3292253 .7593584

------------------------------------------------------------------------------

. logistic dne1 i.vih

Logistic regression Number of obs = 159

LR chi2(1) = 8.50

Prob > chi2 = 0.0036

Log likelihood = -100.07401 Pseudo R2 = 0.0407

**INFECTION VIH**

------------------------------------------------------------------------------

dne1 | Odds Ratio Std. Err. z P>|z| [95% Conf. Interval]

-------------+----------------------------------------------------------------

vih |

1. Oui | 7.919997 6.410311 2.56 0.011 1.620977 38.69662

_cons | .5050505 .0876245 -3.94 0.000 .3594632 .7096027

------------------------------------------------------------------------------

. logistic dne1 i.hbv

Logistic regression Number of obs = 159

LR chi2(1) = 0.06

Prob > chi2 = 0.8122

Log likelihood = -104.29467 Pseudo R2 = 0.0003

**INFECTION HBV : non significatif**

------------------------------------------------------------------------------

dne1 | Odds Ratio Std. Err. z P>|z| [95% Conf. Interval]

-------------+----------------------------------------------------------------

hbv |

1. Oui | 1.17284 .7830073 0.24 0.811 .3169297 4.340251

_cons | .5684211 .0968734 -3.31 0.001 .4070088 .7938465

------------------------------------------------------------------------------

. logistic dne1 i.hcv

Logistic regression Number of obs = 159

LR chi2(1) = 4.88

Prob > chi2 = 0.0272

Log likelihood = -101.88365 Pseudo R2 = 0.0234

**INFECTION HCV**

------------------------------------------------------------------------------

dne1 | Odds Ratio Std. Err. z P>|z| [95% Conf. Interval]

-------------+----------------------------------------------------------------

hcv |

1. Oui | 3.88 2.470159 2.13 0.033 1.114098 13.51263

_cons | .5154639 .08974 -3.81 0.000 .3664442 .7250847

------------------------------------------------------------------------------

. logistic dne1 i.trai

note: 0.trai != 0 predicts failure perfectly

0.trai dropped and 5 obs not used

note: 4.trai != 0 predicts failure perfectly

4.trai dropped and 2 obs not used

note: 5.trai omitted because of collinearity

Logistic regression Number of obs = 152

LR chi2(3) = 0.67

Prob > chi2 = 0.8799

Log likelihood = -100.71862 Pseudo R2 = 0.0033

**TYPE DE TRAITEMENT : Non significatif**

---------------------------------------------------------------------------------

dne1 | Odds Ratio Std. Err. z P>|z| [95% Conf. Interval]

----------------+----------------------------------------------------------------

trai |

0. Aucun | 1 (empty)

1. ADO | 1.032558 .7796345 0.04 0.966 .2350789 4.535399

2. RegimAlim | 1.147287 .7725561 0.20 0.838 .306544 4.293893

3. Insul+Regim | 1.529716 .7990923 0.81 0.416 .5494908 4.258543

4. Insuline | 1 (empty)

5. ADO+Regim | 1 (omitted)

|

_cons | .5810811 .1114242 -2.83 0.005 .3990396 .8461696

---------------------------------------------------------------------------------

. logistic dne1 i.irc

note: 0.irc != 1 predicts success perfectly

0.irc dropped and 4 obs not used

note: 1.irc omitted because of collinearity

note: 2.irc omitted because of collinearity

Logistic regression Number of obs = 155

LR chi2(0) = -0.00

Prob > chi2 = .

Log likelihood = -100.19858 Pseudo R2 = -0.0000

-------------------------------------------------------------------------------

dne1 | Odds Ratio Std. Err. z P>|z| [95% Conf. Interval]

--------------+----------------------------------------------------------------

irc |

1. Oui | 1 (empty)

2. NeSaisPas | 1 (empty)

|

_cons | .5346535 .0901323 -3.71 0.000 .3842166 .7439927

-------------------------------------------------------------------------------

. logistic dne1 i.hta

Logistic regression Number of obs = 159

LR chi2(1) = 0.48

Prob > chi2 = 0.4868

Log likelihood = -104.08108 Pseudo R2 = 0.0023

**HTA : non significatif**

------------------------------------------------------------------------------

dne1 | Odds Ratio Std. Err. z P>|z| [95% Conf. Interval]

-------------+----------------------------------------------------------------

hta |

1. Oui | 1.263158 .4255614 0.69 0.488 .6526578 2.444724

_cons | .5 .1305582 -2.65 0.008 .2997142 .8341281

------------------------------------------------------------------------------

. logistic dne1 i.alc

Logistic regression Number of obs = 159

LR chi2(1) = 1.77

Prob > chi2 = 0.1833

Log likelihood = -103.43757 Pseudo R2 = 0.0085

**ALCOOLISME CHRONIQUE : non significatif**

------------------------------------------------------------------------------

dne1 | Odds Ratio Std. Err. z P>|z| [95% Conf. Interval]

-------------+----------------------------------------------------------------

alc |

1. Oui | 2.173203 1.266546 1.33 0.183 .6934552 6.810548

_cons | .5368421 .0931915 -3.58 0.000 .3820189 .7544115

------------------------------------------------------------------------------

. logistic dne1 i.tab

Logistic regression Number of obs = 159

LR chi2(1) = 0.66

Prob > chi2 = 0.4158

Log likelihood = -103.99183 Pseudo R2 = 0.0032

**TABAGISME : Non significatif**

------------------------------------------------------------------------------

dne1 | Odds Ratio Std. Err. z P>|z| [95% Conf. Interval]

-------------+----------------------------------------------------------------

tab |

1. Oui | .4254386 .480925 -0.76 0.450 .0464109 3.899901

_cons | .5876289 .0980709 -3.19 0.001 .4236858 .8150089

------------------------------------------------------------------------------

. logistic dne1 i.tbc

Logistic regression Number of obs = 159

LR chi2(1) = 0.47

Prob > chi2 = 0.4910

Log likelihood = -104.08575 Pseudo R2 = 0.0023

**Traitement antituberculeux ; non significatif**

------------------------------------------------------------------------------

dne1 | Odds Ratio Std. Err. z P>|z| [95% Conf. Interval]

-------------+----------------------------------------------------------------

tbc |

1. Oui | 1.781819 1.485499 0.69 0.488 .347711 9.130798

_cons | .5612245 .0945558 -3.43 0.001 .4033901 .7808148

------------------------------------------------------------------------------

. logistic dne1 i.ins

Logistic regression Number of obs = 159

LR chi2(1) = 15.37

Prob > chi2 = 0.0001

Log likelihood = -96.638832 Pseudo R2 = 0.0737

**INSTABILITE A MARCHE**

------------------------------------------------------------------------------

dne1 | Odds Ratio Std. Err. z P>|z| [95% Conf. Interval]

-------------+----------------------------------------------------------------

ins |

1. Oui | 3.841463 1.370958 3.77 0.000 1.908606 7.731739

_cons | .2666667 .0750309 -4.70 0.000 .1536278 .4628792

------------------------------------------------------------------------------

. logistic dne1 tai

Logistic regression Number of obs = 159

LR chi2(1) = 1.21

Prob > chi2 = 0.2714

Log likelihood = -103.71796 Pseudo R2 = 0.0058

**TAILLE : Non significatif**

------------------------------------------------------------------------------

dne1 | Odds Ratio Std. Err. z P>|z| [95% Conf. Interval]

-------------+----------------------------------------------------------------

tai | 18.22979 48.25995 1.10 0.273 .101715 3267.22

_cons | .0044001 .0195757 -1.22 0.223 7.19e-07 26.93934

------------------------------------------------------------------------------

. logistic dne1 imc

Logistic regression Number of obs = 159

LR chi2(1) = 2.49

Prob > chi2 = 0.1143

Log likelihood = -103.07608 Pseudo R2 = 0.0120

------------------------------------------------------------------------------

dne1 | Odds Ratio Std. Err. z P>|z| [95% Conf. Interval]

-------------+----------------------------------------------------------------

imc | .7401573 .1438317 -1.55 0.122 .505724 1.083264

_cons | .9820162 .3710975 -0.05 0.962 .4682256 2.059596

------------------------------------------------------------------------------

. logistic dne1 i.imc1

Logistic regression Number of obs = 159

LR chi2(1) = 1.13

Prob > chi2 = 0.2881

Log likelihood = -103.75861 Pseudo R2 = 0.0054

**IMC > 25 : non significatif**

------------------------------------------------------------------------------

dne1 | Odds Ratio Std. Err. z P>|z| [95% Conf. Interval]

-------------+----------------------------------------------------------------

imc1 |

>=25 | .7025761 .2334848 -1.06 0.288 .3662826 1.347629

_cons | .7 .1724819 -1.45 0.148 .4318766 1.134583

------------------------------------------------------------------------------

. logistic dne1 hba1c

Logistic regression Number of obs = 159

LR chi2(1) = 23.35

Prob > chi2 = 0.0000

Log likelihood = -92.646863 Pseudo R2 = 0.1119

**HbA1C général**

------------------------------------------------------------------------------

dne1 | Odds Ratio Std. Err. z P>|z| [95% Conf. Interval]

-------------+----------------------------------------------------------------

hba1c | 2.139061 .3664785 4.44 0.000 1.528937 2.992656

_cons | .0005396 .0008587 -4.73 0.000 .0000239 .0122056

------------------------------------------------------------------------------

. logistic dne1 i.hba1c1

note: 1.hba1c1 != 0 predicts failure perfectly

1.hba1c1 dropped and 11 obs not used

note: 3.hba1c1 omitted because of collinearity

Logistic regression Number of obs = 148

LR chi2(1) = 16.57

Prob > chi2 = 0.0000

Log likelihood = -90.813111 Pseudo R2 = 0.0836

**HbA1C entre 7,6-9**

------------------------------------------------------------------------------

dne1 | Odds Ratio Std. Err. z P>|z| [95% Conf. Interval]

-------------+----------------------------------------------------------------

hba1c1 |

7.2-7.5 | 1 (empty)

7.6-9 | .2432433 .0868743 -3.96 0.000 .1207909 .4898324

9.1-12.5 | 1 (omitted)

|

_cons | 1.37037 .3468528 1.24 0.213 .8344328 2.250529

------------------------------------------------------------------------------

. logistic dne1 gly

Logistic regression Number of obs = 159

LR chi2(1) = 12.33

Prob > chi2 = 0.0004

Log likelihood = -98.158669 Pseudo R2 = 0.0591

**GLYCEMIE**

------------------------------------------------------------------------------

dne1 | Odds Ratio Std. Err. z P>|z| [95% Conf. Interval]

-------------+----------------------------------------------------------------

gly | 1.824198 .3307151 3.32 0.001 1.278659 2.602491

_cons | .1702257 .0693161 -4.35 0.000 .0766329 .3781248

------------------------------------------------------------------------------

. logistic dne1 creat

Logistic regression Number of obs = 159

LR chi2(1) = 37.31

Prob > chi2 = 0.0000

Log likelihood = -85.670193 Pseudo R2 = 0.1788

**CREATININE général**

------------------------------------------------------------------------------

dne1 | Odds Ratio Std. Err. z P>|z| [95% Conf. Interval]

-------------+----------------------------------------------------------------

creat | 1.307686 .0786013 4.46 0.000 1.162359 1.471183

_cons | .0178324 .0139422 -5.15 0.000 .0038521 .08255

------------------------------------------------------------------------------

. logistic dne1 i.creat1

Logistic regression Number of obs = 159

LR chi2(1) = 27.35

Prob > chi2 = 0.0000

Log likelihood = -90.648673 Pseudo R2 = 0.1311

**CREAT1 > 12**

------------------------------------------------------------------------------

dne1 | Odds Ratio Std. Err. z P>|z| [95% Conf. Interval]

-------------+----------------------------------------------------------------

creat1 |

12.1-56.0 | 6.090535 2.205296 4.99 0.000 2.995349 12.38407

_cons | .2432432 .0639267 -5.38 0.000 .1453237 .4071412

------------------------------------------------------------------------------

. logistic dne1 i.imc1

Logistic regression Number of obs = 159

LR chi2(1) = 1.13

Prob > chi2 = 0.2881

Log likelihood = -103.75861 Pseudo R2 = 0.0054

**IMC > 25 : Non significatif**

------------------------------------------------------------------------------

dne1 | Odds Ratio Std. Err. z P>|z| [95% Conf. Interval]

-------------+----------------------------------------------------------------

imc1 |

>=25 | .7025761 .2334848 -1.06 0.288 .3662826 1.347629

_cons | .7 .1724819 -1.45 0.148 .4318766 1.134583

------------------------------------------------------------------------------

. logistic dne1 dn4

Logistic regression Number of obs = 79

LR chi2(1) = 8.94

Prob > chi2 = 0.0028

Log likelihood = -50.12952 Pseudo R2 = 0.0819

------------------------------------------------------------------------------

dne1 | Odds Ratio Std. Err. z P>|z| [95% Conf. Interval]

-------------+----------------------------------------------------------------

dn4 | 1.877107 .4330959 2.73 0.006 1.194251 2.950412

_cons | .0448452 .0542066 -2.57 0.010 .0041959 .4792969

------------------------------------------------------------------------------
